# The incidence of congenital anomalies in newborns before and during the Covid-19 pandemic

**DOI:** 10.1186/s13052-022-01368-6

**Published:** 2022-09-15

**Authors:** Mohammad Heidarzadeh, Mahshid Taheri, Zohreh Mazaheripour, Fariba Abbasi-Khameneh

**Affiliations:** 1 Pediatric Health Research Center, Tabriz University of Medical Sciences, Tabriz, Iran; 2 Department of Hospital Management and Clinical Services Excellence, Ministry of Health and Medical Education, Tehran, Iran

**Keywords:** Covid-19, Pandemic, Congenital anomaly, Central nervous system

## Abstract

**Background:**

Pregnant women are one of the most vulnerable groups in the Covid-19 pandemic. Due to the lack of knowledge about fetal and perinatal complications following Covid-19 infection, the association of Covid-19 pandemic and congenital anomalies in babies conceived and born during this pandemic is unclear. Current study aimed to investigate the association between the Covid-19 pandemic and congenital birth anomalies in Iran. The population of newborns whose embryonic period coincided with the Covid-19 crises were compared with a similar group born during the pre-Covid-19 period.

**Methods:**

This is a retrospective comparative analysis of congenital birth anomalies in Iran; desired data was extracted from national birth registry database. All registered congenital anomalies in hospital births were compared between two time periods: During Covid-19 (1st November 2020- 28th February 2021) and Before Covid-19 (1st November 2019-29th February 2020). Incidence of congenital anomalies at birth were compared and analyzed between these two time periods.

**Results:**

The incidence of congenital birth anomalies are significantly increased during Covid-19 pandemic compared with before Covid-19 (*P* value < 0.00001). The number of all types of anomalies has increased in the current pandemic, but the congenital anomalies of the central nervous system (*P* value = 0.04) and Genitourinary (*P* value = 0.03) have a larger contribution than before.

**Conclusion:**

Covid-19 pandemic are associated with congenital anomalies at birth. There are several factors in the Covid-19 pandemic which can affect fetal development in the first trimester of pregnancy. Possible reasons include vertical transmission of Covid-19 infection; maternal fever, stress and anxiety; insufficient preconception and prenatal care; neglect of fetal screening; and poverty imposed by this pandemic.

## Introduction

Covid-19 pandemic has broad impacts on a person’s reproductive health, perinatal period, and childbirth choices [1, 2]. The childbearing plans of many couples have been postponed and the majority of women are concerned about receiving inadequate perinatal care. Pandemic-related worries have a direct impact on women's physical and mental health and their potential pregnancies [1, 3]. The mother's stress can play an important role in the development of the fetus [3], but more importantly, there is a possibility of vertically transmitted covid-19 infection and subsequent birth abnormalities. There is no reliable evidence for transplacental transmission of Covid-19 during the first or early second trimester of pregnancy, however, current limited data do not indicate maternal-to-fetal transmission in the third trimester [4].

Collecting and analyzing data on association Covid-19 crisis and perinatal complications, especially congenital anomalies, is a research priority [4]. In this study we aimed to investigate the association of the covid-19 pandemic and congenital birth anomalies in Iran. In particular, the population of newborns whose embryonic period coincided with the Covid-19 crises have been studied.

## Methods

This is a retrospective comparative analysis of birth prevalence for congenital anomalies in Iran. The population-based data were collected from the “Iranian Maternal and Neonatal” (IMaN) Network. This online birth registry database is a national network that all the hospitals register their information about details of childbirth, administered by the Iranian Ministry of Health. It records all birth-related data electronically (demographics, maternal characteristics, medical history, obstetric interventions, pregnancy complications, and perinatal outcomes) across the country since 2013. The national accreditation program confirmed that there is acceptable validity and reliability in the recent years with more than 95% of the registration rate [5]. Almost from the beginning of online birth registry in Iran (2013), surveillance of birth defects has been started. Obviously, more details have been added to this monitoring over time and its accuracy has increased. Currently, the hospital user completes the online birth registry form according to the healthcare providers’ examinations that are recorded in the birth documents.

All registered congenital anomalies in hospital births were compared between two time periods; one of these time periods was chosen to include births of newborns who their entire fetal period, especially the first trimester of pregnancy coincided with the Covid-19 outbreak in Iran (1st November 2020- 28th February 2021). The second time period includes the same four months of the past year, before the onset of Covid-19 in Iran (1st November 2019- 29th February 2020). Currently, the history of maternal Covid-19 infection during pregnancy is not registered in IMaN; given the data available, the method used in this study is the most logic way to find the possible association between the Covid-19 infection and congenital anomalies.

Maternal characteristics that were assessed included maternal age at birth, last educational certificate, parity, and the type of delivery. Neonatal outcomes were compared between the two selected periods. These outcomes included stillbirth, mortality, and preterm birth. Having at least one birth defect in examinations at birth was considered as a congenital anomaly in the registry. In IMAN online form, there is a question regarding the detection of any abnormality in newborns examination. If the answer is yes, the user has one or more options to select the kind of this congenital abnormality. These congenital defects are categorized into 10 general groups in IMaN network: Central nervous system (CNS), musculoskeletal, gastrointestinal tract, eye-face-ear, heart, skin, genitourinary, respiratory, chromosomal disorder, and unclassified abnormalities. In this study, the same groupings were used and the prevalence of each congenital anomaly type was compared between the Covid-19 pandemic and the pre-Covid-19 time. It should be noted that some newborns had more than one type of congenital anomaly.

Data analysis was performed with the statistical software StataCorp (version 16.1). Data are shown as frequencies (percentages). The Chi-squared test with 95% confidence intervals is used to compare groups’ proportions. The significance level was considered as a *p*-value less than 0.05.

## Results

Total number of newborns born in Covid-19 pandemic period is 347839 vs 395,728 newborns born in the same time period before Covid-19. Table 1 presents demographic characteristics of the mothers who have given birth during these time periods. Variables associated with neonatal anomalies include maternal afge and maternal education before and during Covid-19 pandemic (*P* value < 0.00001), and the type of delivery in the pre-Covid-19 time (*P* = 0.01).

**Table 1:** Demographic characteristics of Iranian women who have given birth during selected periods

**Characteristics**	**During Covid-19:** 1^st^ November 2020- 28^th^ February 2021			**Before Covid-19:** 1^st^ November 2019- 29^th^ February 2020		
	Mother of newborn without anomaly ***N* = 345,827**	Mother of newborn with anomaly ***N* = 2012**	*P* value* (Chi-square)	Mother of newborn without anomaly ***N* = 393,860**	Mother of newborn with anomaly ***N* = 1868**	*P* value* (Chi-square)
**Age**						
< 18	13,443 (3.89)	91 (4.52)	< 0.00001*	15,392 (3.91)	82 (4.39)	< 0.00001*
18–35	260,753 (75.40)	1429 (71.02)		301,030 (76.43)	1358 (72.69)	
> 35	71,631 (20.71)	492 (24.46)		77,438 (19.66)	428 (22.92)	
**Education**						
< high school	118,870 (34.37)	884 (43.94)	< 0.00001*	135,260 (34.34)	791 (42.34)	< 0.00001*
High school	149,718 (43.29)	780 (38.76)		171,128 (43.45)	739 (39.56)	
≥ college	77,239 (22.34)	348 (17.30)		87,472 (22.21)	338 (18.10)	
**Parity**						
primiparous	123,507 (35.71)	710 (35.29)	0.69	151,436 (38.45)	736 (39.40)	0.39
multiparous	222,320 (64.29)	1302 (64.71)		242,424 (61.55)	1132 (60.60)	
**Type of delivery**						
NVD	161,801 (46.79)	912 (45.33)	0.19	190,441 (48.35)	852 (45.61)	0.01*
CS	184,026 (53.21)	1100 (54.67)		203,419 (51.65)	1016 (54.39)	

^*^ The significance level was set at *P* value < 0.05

2012 (0.58) of newborns born during Covid-19 pandemic had congenital abnormalities and 1868 (0.47) newborns with congenital abnormalities were born in the same time period pre-Covid-19. The incidence of congenital anomalies in the Iran was higher during Covid-19 than before Covid-19; the chi-square value is 40.36 (*P* value < 0.00001). Although the prevalence of congenital anomalies has increased in the Covid-19 period, neonatal outcomes among abnormal newborns was no significant difference compared to the pre-Covid-19 abnormal babies (Table 2).

**Table 2:** Neonatal outcomes among newborns with congenital anomalies (during vs before Covid-19 pandemic)

**Neonatal outcomes in congenital anomalies**	**Abnormal newborns born in Covid-19 pandemic period:** 1^st^ November 2020- 28^th^ February 2021 ***N* = 2012**	**Abnormal newborns born in the same time period before Covid-19:** 1^st^ November 2019- 29^th^ February 2020 ***N* = 1868**	**Chi-square value**	*P* value*
**Stillbirth**	166 (8.25%)	154 (8.24%)	0.00	0.99
**Neonatal mortality**	125 (6.21%)	93 (4.97%)	2.78	0.95
**Preterm birth**	650 (32.30%)	560 (29.97%)	2.45	0.11

^*^ The significance level was set at *P* value < 0.05

As shown in Table 3, some types of anomalies have increased significantly during the Covid-19 pandemic. CNS and genitourinary abnormalities in newborns of women who have spent their entire pregnancy (trimester one, two and three) during Covid-19 pandemic have increased significantly compared to the same period last year.

**Table 3:** Share of different types of congenital anomalies in total abnormal newborns outcomes (during vs before Covid-19 pandemic)

Type of congenital anomaly*	**Newborns born in Covid-19 pandemic period:** 1^st^ November 2020- 28^th^ February 2021 **N (**% of total abnormal newborns**)**	**Newborns born in the same time period before Covid-19:** 1^st^ November 2019- 29^th^ February 2020 **N (**% of total abnormal newborns**)**	**Chi-square value**	*P* value**
**CNS**	247 (12.27)	191 (10.22)	4.07	0.04**
**Musculoskeletal**	554 (27.50)	527 (28.21)	0.22	0.63
**Gastrointestinal tract**	110 (5.46)	104 (5.56)	0.01	0.89
**Eye, face, ear**	241 (11.97)	221 (11.83)	0.02	0.88
**Heart**	144 (7.15)	116 (6.20)	1.39	0.23
**Skin**	83 (4.12)	72 (3.85)	0.18	0.66
**Genitourinary**	319 (15.85)	251 (13.43)	4.51	0.03**
**Respiratory**	50 (2.48)	37 (1.98)	1.12	0.28
**Chromosomal disorder**	82 (4.07)	77 (4.12)	0.00	0.94
**Unclassified**	294 (14.61)	285 (15.25)	0.31	0.57

^*^ Some newborns had more than one type of anomaly, so the sum of the values in each column is greater than the total number of abnormal newborns

^**^Show significant association with *P* values < 0.05

Subtypes of CNS anomalies in IMAN were: anencephaly, encephalocele, microcephaly, hydrocephaly, Arnold chiari, spina bifida, myelomeningocele, holoprosencephaly, Dandy Walker, and others. Spina bifida was the most common abnormality in both time periods, but there was a statistically significant increase in its prevalence during Covid-19 (33.6% vs 24%; Chi-square = 4.69, *p*-value = 0.03). Another significant difference was detectable in anencephaly subtype (26% vs 16%; Chi-square = 5.94, *p*-value = 0.01). There was no significant difference in other subgroups.

In the current study, genitourinary anomalies include renal agenesis, hydronephrosis, cryptorchidism, epispadias, hypospadias, ectopic kidney, gender ambiguity, polycystic kidney, bladder exstrophy. Hydronephrosis as the most prevalent subtype, showed a significant increase in the Covid-19 pandemic (58% vs 43.5%; Chi-square = 11.93, *p*-value = 0.0005). Polycystic kidney prevalence was 25% during Covid-19 vs 17% before pre-Covid-19 (Chi-square = 5.41, *p*-value = 0.01). The last significant difference was seen in the renal agenesis subgroup (20% vs 13%; Chi-square = 5.15, *p*-value = 0.02).

## Discussion

We found statistical evidence of association between the Covid-19 pandemic and increased incidence of congenital anomalies. The types of abnormalities that their share of total have significantly increased in this pandemic include CNS and genitourinary anomalies. This finding contradicts the predictions available from the SARS-CoV epidemic experience [6]. Iranian women who spent their first trimester of pregnancy in the Covid-19 crises were more likely to give birth to a baby with a congenital anomaly. In our opinion, this does not mean that the viral infection has a direct impact on the abnormal embryo development but other variables caused by this pandemic can also be associated with congenital anomalies. Maternal fever during first trimester could be associated with higher risk of anomalies in the genital system [7]. Another factor is chronic maternal stress during Covid-19 pandemic and its impact on fetal development [3]. Other possible causes include decreased quantity, quality, and routine referrals of prenatal care [8], poverty due to the Covid-19 pandemic and lockdowns [9], and insufficient referrals for fetal screening and diagnosis [10]. Our periodic monitoring of the validity of IMAN records has not shown any visible difference between 2020 and 2021. Therefore, it is unlikely that the detected difference between the two time periods was associated with the better ascertainment in the 2021 records.

Underlying variables including maternal age and education were significantly associated with congenital anomalies. Advanced maternal age and lower education status are factors that have increased the risk of major congenital anomalies [11, 12]. During the Covid-19 pandemic, there was an increase in caesarean section in the general population; while in pre-Covid-19 period, women with abnormal fetuses were more likely to undergo a caesarean section compared with general population.

The overall reported percentage of congenital abnormalities in this study was lower than the international rate (1.5–3%) [13]. The high prevalence of prenatal screening methods in Iran has led to a significant reduction in the congenital abnormality rate at delivery; most families prefer to abort their abnormal baby during the second trimester. Government health policy regarding folic acid supplementation and fortification of flour, consider an important factor in the reduction of NTDs [14]. It is worthwhile to mention that the diagnosis of CA at delivery is slightly less accurate than in the later months of life [15].

The information used in this study is affected by the accuracy of registered data and the registered precent in the IMaN network; Data validity of IMaN has reached in acceptable level in recent years. One of the main limitation is lack of information about maternal Covid-19 infection during pregnancy. Therefore, it was not possible for us to assess a direct correlation between Covid-19 infection and congenital anomalies. For more reliable information, cohort studies are recommended to accurate risk assessment of covid-19 infected women during their first trimester of pregnancy for the congenital anomalies in their offspring. Assessment of confounding variables such as insufficient prenatal visits and screenings during this pandemic and their effects on congenital abnormalities at delivery would be an interesting topic for future works.

## Conclusion

Covid-19 pandemic are associated with congenital anomalies at birth. There are several factors in the Covid-19 pandemic which can affect fetal development in the first trimester of pregnancy. Possible reasons include vertical transmission of Covid-19 infection; maternal fever, stress and anxiety; insufficient preconception and prenatal care; neglect of fetal screening; and poverty imposed by this pandemic.

## Data Availability

The data are stored on a secured server within the IMaN network; they can only be accessed under regulations: owns and permissions.

## Abbreviations

IMaN: Iranian Maternal and Neonatal
CNS: Central Nervous System
